# Application of endoscopy to treat mandibular keratocystic odontogenic tumors

**DOI:** 10.1590/1414-431X20176209

**Published:** 2017-07-10

**Authors:** Z. Gao, Q.W. Ni, W. Gao, Y.P. Liu, Q. Zhang

**Affiliations:** 1 Department of Oral and Maxillofacial Surgery, General Hospital of Xinjiang Military Region, Urumqi, China; 2 State Key Laboratory of Military Stomatology & National Clinical Research Center for Oral Diseases & Shaanxi Clinical Research Center for Oral Diseases, Department of Oral and Maxillofacial Surgery, School of Stomatology, Fourth Military Medical University, Xi'an, China

**Keywords:** Mandibular KCOT, Endoscopic assisted surgery, 3D reconstruction, Minimally invasive surgical procedures, Treatment feasibility

## Abstract

The aim of this study was to evaluate the feasibility of endoscopy to remove keratocystic odontogenic tumors (KCOTs) with virtual 3D mandibular images. Fifteen patients (mean age, 40.27±14.58 years) who underwent endoscopic mandibular KCOT enucleation between May 2009 and October 2009 were included. Virtual 3D mandibular reconstructions derived from computed tomography (CT) imaging were generated for all patients. Recurrence and pathological fracture were evaluated as the primary outcome variables at 1 and 12 months after operation. Secondary infection and inferior alveolar nerve injury were evaluated as the secondary outcome variables at 1 and 6 months after operation. None of the 15 patients exhibited signs of recurrence or pathological fracture after operation. During long-term follow-up, no symptoms of inferior alveolar nerve injury or secondary infection were observed and no signs of recurrence were found in any of the patients. Endoscopy helps surgeons to remove mandibular KCOTs with small incisions. Moreover, endoscopy can provide clear and magnified views and help to avoid damage to the inferior alveolar neurovascular bundle. Therefore, under the support of preoperative virtual 3D mandibular images, the application of endoscopy to remove the tumors should be considered to be a treatment option for KCOTs.

## Introduction

In 2005, the World Health Organization (WHO) defined keratocystic odontogenic tumor (KCOT) as a benign uni- or multi-cystic intraosseous tumor of odontogenic origin with a characteristic lining of parakeratinized stratified squamous epithelium and with the potential for aggressive infiltrative behavior. KCOTs tend to occur in the mandibular ramus and mandibular molar regions. Approximately 60% of KCOTs occur at the mandibular body and mandibular ramus (1), and 25 to 40% of KCOTs involve the teeth in the region of the lesion (2). In China, KCOTs account for 35.8% of all odontogenic tumors, ranking them third among odontogenic tumors (3). Based on X-ray images, KCOTs are categorized into four types: a) unilocular, which is the most common and accounts for 48.8% of tumors; b) multilobular, which includes many lobules that are not completely separated by the bony septum and accounts for 22.0% of tumors; c) scalloped, in which the mandible is eroded by the KCOT, producing a scallop-edged image containing many crenate shapes and which accounts for 20.7% of tumors, and d) multilocular, which accounts for 8.5% of tumors (4). Multilocular tumors are particularly difficult to treat due to their large volume and high postoperative recurrence rate, which exceeds that of unilocular tumors (P=0.0350) (5,6).

At present, many treatments are available for KCOTs, such as enucleation, curettage, cryosurgery, resection, marsupialization and decompression. But these treatments have certain disadvantages, including high recurrence rates, long treatment periods, severe damage to the surrounding tissue, various postoperative complications, and poor control during the treatment period. Three-dimensional (3D) reconstruction can be applied to reconstruct a virtual mandibular image and directly depict the position, local invasion and approximate volume of the tumor. Furthermore, the reconstructed image could determine whether the tumor has contact with the inferior alveolar nerve (IAN) or is separate from the IAN. Endoscopy provides clear views, facilitating smaller incisions. Over the past five years, we have attempted to use a method that combines endoscopy with 3D reconstruction to treat KCOTs.

Therefore, the specific aims of the present study were: 1) to evaluate the effect of intraoperative visualization with endoscopy, and 2) to evaluate the outcome of using endoscopy to remove mandibular KCOTs.

## Material and Methods

### Study population

This study was approved by the Ethics Committee of the General Hospital of Xinjiang Military Region (protocol IRB-REV-2009006). Written informed consent was obtained to publish the clinical photographs from the patients.

To address the research purpose, the investigators designed and implemented a retrospective study at the Department of Oral and Maxillofacial Surgery, General Hospital of Xinjiang Military Region. The study population included adult patients who underwent mandibular KCOT enucleation involving endoscopy with preoperative virtual 3D mandibular images between May and October 2009. Basic information (gender and age), clinical data (diagnosis, affected side, lesion type, lesion volume and relation with IAN) and the follow-up duration were collected.

Regarding the exclusion criteria, patients with infections or malignant transformation and those with nevoid basal cell carcinoma syndrome were not considered in the study. The multilocular KCOT was not included in the study because this type of lesion is not suitable for operation using endoscopy. Patient exclusion was based on preoperative imaging exams and fine-needle aspiration biopsy.

### Outcome variables

The primary outcome measures were recurrence and pathological fracture. The secondary outcome measures were secondary infection and IAN injury during the 6 months of follow-up after surgery.

### Recurrence and pathological fracture

Recurrence and pathological fracture were evaluated by conducting postoperative panoramic radiographic examinations at 1 and 12 months after the operation. These exams were considered to be direct data. Furthermore, swelling and malocclusion on the surgical side, which indirectly represented recurrence and pathological fracture, were assessed during long-term follow-up.

### Secondary infection and IAN injury

Secondary infection and IAN injury were evaluated by local investigation in the operative field at 1, 3, and 6 months after the operation. Local symptoms, including incisional swelling and suppuration, were examined. The patients were asked to provide their subjective opinion regarding lower lip numbness, pain and swelling on the surgical side.

### Data collection

#### Preoperative data collection and design

**Figure 1.**
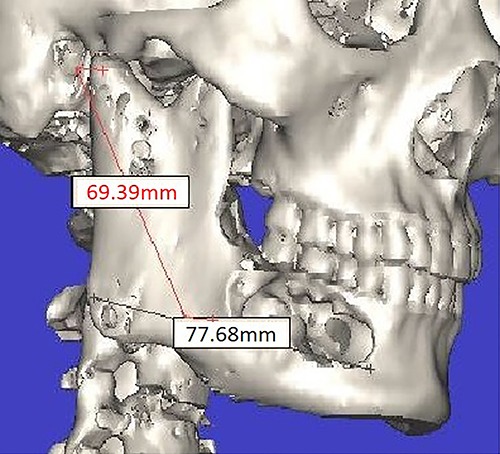
Reverse measurement.

Patients underwent a preoperative cranial-maxillofacial computed tomography (CT) scan (slice thickness, 0.5 mm) (SIEMENS, Germany). The data were stored and imported into SimPlant Pro 11.04 software (Materialise Company, Belgium) for 3D cranial-maxillofacial reconstruction (reconstructive thickness, 1 mm). The position, local invasion and approximate volume of the tumor were subsequently measured on the 3D virtual mandibular images and recorded in SimPlant software (Figure 1). Next, by combining the image of 3D reconstruction with a panoramic radiograph, we documented the bony septum of the tumor and established the direction and position of the endoscope access. Generally, the endoscope was inserted at the defect of the buccal cortex and advanced from the bottom to the posterior side between the tumor and the bone tissue at an angle of 30°–70°.

#### Endodontic evaluation and treatment

Pulp testing was done before surgical treatment. All 15 patients received electric pulp testing for the teeth adjacent or involved by the lesions (no patient used an artificial cardiac pacemaker). The teeth were adequately dried and isolated before testing. The healthy teeth of contralateral side were tested as controls to observe a baseline normal response. The probe with conductive medium was placed on the tooth, and the readings were recorded. Compared with the controls, the pulp vitality was normal if the difference of readings was under 20; the pulp vitality was decreased if the difference of readings was over 20. The pulp vitality was negative if the reading was over 80 with no response. The teeth with decreased and negative pulp vitalities underwent root canal treatment before surgery. Two weeks after surgery, the teeth, whose pulp vitalities were normal before surgery, underwent pulp testing again. If the pulp vitalities were decreased or negative, root canal treatment was carried out. Likewise, root canal treatment was done if patients had pulp symptoms.

#### Operative procedure

All of the patients received general anesthesia with nasal intubation. An incision was made on the oral vestibular mucosa at a location corresponding to the position of the tumor. We removed cortical bone on the tumor surface and formed an access point or used the cortical bone defect as an access point for the endoscope (Olympus, Japan). At the beginning of the procedure, the tumor tissue was separated from the mandible around the access point. Next, the endoscope was placed between the tumor and the bone. First, we carefully separated the buccal and mesial parts of the tumor until we reached the bottom of the bony cavity and then separated the lingual and distal parts until the tumor was completely separated.

**Figure 2.**
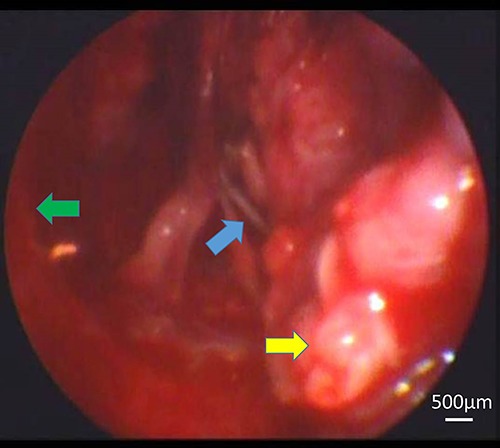
Endoscopy showing tumor adhesion to the inferior alveolar nerve (blue arrow), teeth (yellow arrow) and bone wall (green arrow) (magnification 10×).

**Figure 3.**
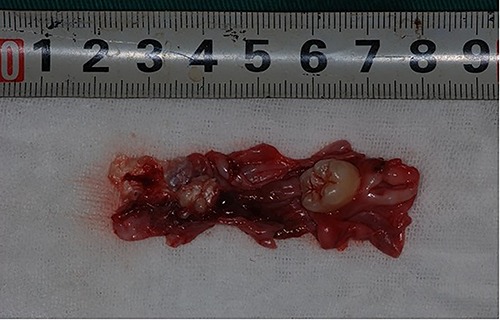
Dissected keratocystic odontogenic tumor.

**Figure 4.**
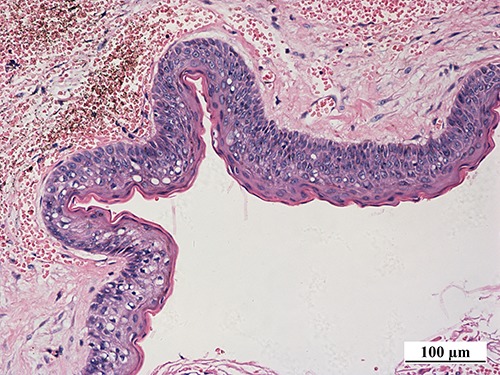
Photomicrograph of a mandibular keratocystic odontogenic tumor in which the epithelial tumor had a wave-shaped stratified squamous epithelium (magnification 200×).

A clear visualization of the tumor inside of the mandible could be obtained using the endoscope (Figure 2). For multilobular and scalloped KCOT, most of the incomplete and thin bony septa were removed during surgery to ensure that the endoscope could enter without being blocked by the septum and that sufficient space was available for the operation. Endoscopic instruments were used to precisely dissect the tumor from the mandible through an endoscopic working channel, especially near important structures, such as the areas adjacent to the inferior alveolar canal, thereby enabling precise tumor separation (Figure 3). After the tumor was removed, an electrotome (ERBE, Germany) was used to cauterize the inner surface of the bony cavity. The carbonized tissues were scraped three times to stop bleeding and to eliminate tumor remnants and potential satellite cysts. Artificial bone (granulated coralline hydroxyapatite bone, YHJ, Beijing, China) was packed inside the bony cavity under endoscopic guidance. Postoperative histological examination confirmed the diagnosis of KCOT (Figure 4).

### Data analyses

Four variables, including recurrence, pathological fracture, secondary infection and inferior alveolar nerve injury, were used to assess the clinical efficacy of the combination treatment of 3D reconstruction and endoscopy for the enucleation of mandibular KCOT.

Local examination of the operative field was performed at 1, 3, and 6 months following the operation. Secondary infection was considered if incisional swelling or suppuration was found. IAN injury was considered if lower lip numbness and swelling on the surgical side were reported. Postoperative panoramic radiographic examinations of each patient were performed to evaluate recurrence and pathological fracture at one month and one year after surgery. The patients were followed up for an extended period of time with a median duration of 52 months (range, 44 to 60 months).

## Results

A total of 15 patients were included in this study: 8 males and 7 females. The mean patient age was 40.27±14.58 years (range, 18 to 76 years). The left side was affected in 10 patients, and the right side was affected in 5 patients. All 15 patients were diagnosed with mandibular KCOTs. Unilocular, multilobular and scalloped lesion types were included (10, 2, and 3 patients, respectively). The tumors volume and the relation with IAN were also recorded (Table 1)

**Table 1.** Patient information.

Patient No.	Gender	Age (year)	Preoperative diagnosis	Affected side	Lesion type	Lesion volume (cm^3^)	Relation with IAN	Postoperative diagnosis	Follow-up duration (month)
1	Female	51	KCOT	Left	Unilocular	71.632	Contact	KCOT	50
2	Female	39	KCOT	Right	Unilocular	50.693	Contact	KCOT	57
3	Male	18	KCOT	Right	Unilocular	74.536	Contact	KCOT	51
4	Female	34	KCOT	Left	Unilocular	66.792	Contact	KCOT	49
5	Male	47	KCOT	Left	Multilobular	26.973	Separate	KCOT	60
6	Male	36	KCOT	Right	Scalloped	18.634	Contact	KCOT	54
7	Male	37	KCOT	Left	Unilocular	41.178	Contact	KCOT	56
8	Female	40	KCOT	Left	Unilocular	76.472	Contact	KCOT	52
9	Male	50	KCOT	Left	Scalloped	12.474	Separate	KCOT	58
10	Male	33	KCOT	Left	Unilocular	36.698	Contact	KCOT	46
11	Female	76	KCOT	Left	Scalloped	11.265	Separate	KCOT	52
12	Male	24	KCOT	Right	Unilocular	103.740	Contact	KCOT	50
13	Female	30	KCOT	Left	Unilocular	28.067	Separate	KCOT	44
14	Female	59	KCOT	Left	Multilobular	18.019	Contact	KCOT	54
15	Male	30	KCOT	Right	Unilocular	25.150	Separate	KCOT	53

KCOT: keratocystic odontogenic tumor;

IAN: inferior alveolar nerve.

**Figure 5.**
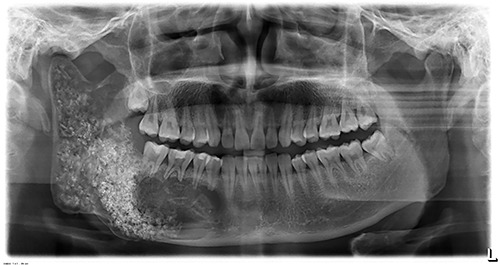
Complete fusion of the bone graft with surrounding bone 1 month after surgery.

**Figure 6.**
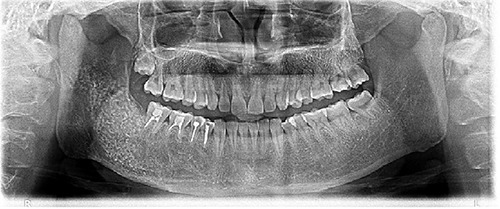
Complete fusion of the bone graft and surrounding bone 1 year after surgery.

None of the patients had pathological fractures after the operation. One month after surgery, a panoramic radiographic examination showed that the outline of the mandible was preserved and that the bone graft (artificial bone) was uniformly distributed in the bony cavity (Figure 5). None of the patients had a secondary infection during the 6 months after surgery. One month after surgery, 9 patients (60.0%) reported lower lip numbness, and 4 (26.7%) patients reported swelling on the surgical side, but their panoramic radiographic exams showed no signs of recurrence. Three months after surgery, 2 patients (13.3%) reported lower lip numbness, and no patient reported swelling on the surgical side. Six months after surgery, no patient reported lower lip numbness. One year after surgery, the panoramic radiographic examinations showed that all of the bone grafts survived. We performed long-term follow-up for all 15 patients (Figure 6). No symptoms of IAN injury and no sign of recurrence were found in any of the patients at long-term follow-up.

## Discussion

There are three major causes of KCOT recurrence, including the presence of surgical remnants, satellite cysts and dental lamina rests in the general area of the original tumor (7). Therefore, the challenge of treatment is the proper cleaning of the cavity and removal of the epithelial residue, residual tumor cells and satellite cysts to prevent the recurrence of cysts. Therapy is generally chosen according to the tumor volume, recurrence status and imaging characteristics (8). For KCOTs, the best treatment is to enucleate the tumor completely, appropriately treat the bony cavity, completely remove residual tumor cells, satellite cysts and any other recurrence risks, completely preserve the bone structure, and perform reconstruction of the bone structure (9). The purpose of this study was to evaluate the possibility of using endoscopy to remove KCOTs with preoperative virtual 3D mandibular images. In this case series, KCOT recurrence was not identified by clinical panoramic radiographic examination during the follow-up period. No secondary infections, pathological fractures or severe IAN injuries occurred. Thus, the application of endoscopy to remove the tumors should be considered for treatment for KCOTs.

Recurrence is the main reason for the failure of the KCOT treatment, and it depends on therapy approach (10); for example, recurrence rates following enucleation, curettage, cryosurgery, marsupialization, Carnoy's solution and decompression are approximately 54.55% (11,12), 19.2% (13), 3% (14), 21.4% (15), 6.7% (12), and 25% (16), respectively. The recurrence rate following our treatment was lower than these treatments and it may be due to two reasons: the small number of cases and not enough follow-up time. On the other hand, different lesion types may lead to different recurrence rates. Further investigations need to be performed for these problems.

Meanwhile, the common treatments for KCOTs have certain disadvantages that are difficult to overcome. Enucleation can easily lead to tumor rupture and tumor tissue remnants, increasing the potential for recurrence. Large incisions and extraoral incisions, which are produced by local or radical resection, can cause secondary infections. Additionally, this procedure results in mandibular defects, tooth loss and dental dysfunction (17). The disadvantages of marsupialization and decompression include the possibility of secondary infection, complex procedures, long treatment periods, a high dependence on patient compliance and the need for a second surgery to completely remove the tumor (18). Our experience also indicates that marsupialization and decompression are not applicable in middle-aged or elderly patients because new bone forms notably slowly, and secondary infection is difficult to control in these patients. Carnoy's solution carries the risk of IAN damage if the tumor is adjacent to this structure (19). The component of Carnoy's solution, chloroform, may increase the KCOT malignant transformation risk (12,20). Liquid nitrogen, which is used in cryosurgery, likely delays the healing of the surgical wound and causes an unpredictable bulge or necrosis of the soft tissues surrounding the surgical region (21–23).

Preoperative 3D reconstruction indicates the location of the KCOT in the mandible and its relationship with the inferior alveolar canal, provides the volume of the KCOT, and provides key data for bone reconstruction, which is performed by packing an artificial bone graft into the bony cavity. These also help to reduce surgical time and aid accurate tumor removal through a smaller incision using an endoscope. The technique is beneficial for postoperative recovery and for preventing postoperative infection. During the operation, endoscopy enables a smaller incision than is required for the more common operations. Through applying endoscopy in mandibular KCOT operation, surgeons can obtain direct and magnified visualization. This visibility makes the operative procedure easier and more direct than traditional procedures and is beneficial for surgeons to operate in regions that contain complex structures and important tissues such as the IAN. The application of endoscopy contributes to remove the tumor completely, to decrease the risk of recurrence and to preserve the complete bone structure to facilitate recovery and reconstruction. Additionally, a rigid endoscope was chosen for these operations considering its manipulability. The bony cavity was packed with artificial bone to prevent secondary infections and postoperative pathological fractures, and to prepare the mandible for subsequent dental implantation, if necessary.

There are some disadvantages of this method. First, application of endoscopy increased the cost. Second, endoscopy was not appropriate in patients with small volume lesions, for example lesions that involve only 1–2 teeth, due to the limited space. Third, this method was not suitable for treatment of the multilocular KCOT. Although these tumors have large volumes, the presence of complete bony septa forming separate cavities makes endoscopic exploration difficult.

Our study had several limitations. Because this is a new treatment method for KCOTs, some patients did not want to attempt the operation. Therefore, a small number of patients were treated and thus it was a small case series. Furthermore, follow-up duration was too short to assess long-term outcomes.

During the surgical procedure, we obtained clear, magnified views using an endoscope. Endoscopy can help to identify and eliminate residual tumor tissues but does not sufficiently magnify the tissue to allow for the direct identification of satellite cysts. Therefore, endoscopy cannot be used to directly identify and eliminate satellite cysts.

In conclusion, under the support of preoperative virtual 3D mandibular images, the application of endoscopy to remove KCOTs should be considered to be a treatment strategy. We will continue to perform this procedure to improve its outcome and will continue to observe the long-term curative effects. In future studies, we will compare the outcomes of this treatment to those of other traditional treatments.
